# Dysfunctional Parenting Styles Are Associated with Mental Disorders and Low Self-Efficacy Beliefs in Brazilian Undergraduate Medical Students

**DOI:** 10.1155/2021/6372922

**Published:** 2021-07-13

**Authors:** Vânia Meira Siqueira-Campos, Mariana Siqueira Campos De Deus, Larissa Arbués Carneiro, Alessandra Vitorino Naghettini, Maria Amélia Dias Pereira, José Miguel De Deus, Délio Marques Conde

**Affiliations:** ^1^Health Sciences Program, School of Medicine, Federal University of Goiás, Goiânia, Goiás, Brazil; ^2^School of Medicine, Federal University of Goiás, Goiânia, Goiás, Brazil; ^3^Institute of Tropical Pathology and Public Health, Federal University of Goiás, Goiânia, Goiás, Brazil; ^4^Department of Pediatrics, School of Medicine, Federal University of Goiás, Goiânia, Goiás, Brazil; ^5^Department of Mental Health, School of Medicine, Federal University of Goiás, Goiânia, Goiás, Brazil; ^6^Department of Gynecology and Obstetrics, School of Medicine, Federal University of Goiás, Goiânia, Goiás, Brazil

## Abstract

**Objective:**

To investigate the association between parental bonding styles and anxiety, depression, suicidal ideation, and self-efficacy beliefs in undergraduate medical students.

**Methods:**

A cross-sectional, self-administered survey involving 315 Brazilian medical students was conducted online. The Parental Bonding Instrument (PBI), the Generalized Anxiety Disorder-7 (GAD-7) scale, the 9-item Patient Health Questionnaire (PHQ-9), the Suicidal Behaviors Questionnaire-Revised (SBQ-R), and the general self-efficacy (GSE) scale were used. The internal consistency of the instruments used in the study was analyzed using Cronbach's alpha. Multiple logistic regression models were applied, and the odds ratios (OR) and respective 95% confidence intervals (CI) were calculated to determine the association between parental bonding styles and anxiety, depression, suicidal ideation, and general self-efficacy beliefs.

**Results:**

In the analysis adjusted for sociodemographic variables, maternal affectionless control was associated with a greater risk of anxiety (OR = 2.48; 95% CI: 1.15-5.33), depression (OR = 7.54; 95% CI: 3.20-17.78), suicidal ideation (OR = 3.62; 95% CI: 1.58-8.27), and low self-efficacy (OR = 3.81; 95% CI: 1.76-8.25), while maternal neglectful parenting was associated with depression (OR = 3.24; 95% CI: 1.17-8.96) and paternal affectionate constraint with suicidal ideation (OR = 3.09; 95% CI: 1.36-7.02).

**Conclusions:**

These findings showed dysfunctional parenting styles to be associated with mental illnesses and low self-efficacy in Brazilian undergraduate medical students. This should be taken into consideration when treating medical students with mental disorders.

## 1. Introduction

The high prevalence of mental disorders in undergraduate medical students has become a health concern worldwide [1]. In Brazil, prevalence studies in medical students have reported 30.1% of anxiety, 29.8% of depression [2], and 7.2% of suicidal ideation [3]. These rates are higher than those found for the general population [4]. Mental disorders may exert a negative impact on the academic performance [5] and the quality of life of medical students [2]. These disorders often persist during professional life, ultimately reflecting on the care doctors will offer to their patients [1].

Sociodemographic characteristics such as sex, skin color, and monthly family income [2], as well as personal issues and health status [6], have been reported as factors associated with anxiety and depression in medical students. Many aspects of medical school, including extensive content with long hours of studying and the overall learning environment [6], are themselves considered stressors, contributing towards rendering those in such environments more prone to mental disorders. Nonetheless, another large proportion of undergraduate medical students manage to maintain their mental health despite being submitted to these same conditions [1, 2]. Therefore, the sociodemographic characteristics of the individuals and aspects of the medical course would appear insufficient to establish any causal inference between mental disorders in medical students.

Therefore, it appears likely that other conditions affect how successfully students tackle the challenges associated with medical school and its constant demand for high performance within narrow margins of error [7]. In this respect, social and emotional skills, developed early in life within the family environment, can contribute positively to academic performance [8]. Conversely, a lack of these skills may constitute a limiting factor in the establishment of goals and in the persistence and effort required to meet them [5]. Self-efficacy, the belief in one's own capacity to successfully execute the actions required to meet different challenges, is one of the factors that have been associated both with well-being [9] and with anxiety [10] and suicidal ideation [11].

It is pertinent, then, to consider the relevant role that parents, as the figures who provide affection, may play in the development of social-emotional skills and internal working models of self during childhood and adolescence [12]. In fact, children whose parents provide a safe foundation and encourage them to move forward towards independence appear to develop a secure attachment and grow up to be self-assured and self-reliant, as well as trusting, cooperative, and helpful towards others [12]. Conversely, adverse parenting has been associated with many forms of mental disorders including anxiety [12, 13], depression [12, 13], and suicidal ideation [8, 14].

Consequently, adverse parenting styles may have an impact on self-efficacy beliefs and mental health in undergraduate medical students. Therefore, the objective of the present study was to investigate the association between parenting styles and anxiety, depression, suicidal ideation, and self-efficacy beliefs in medical students.

## 2. Methods

### 2.1. Sample

This cross-sectional, online, self-administered survey was conducted between September and November 2019 at the School of Medicine, Federal University of Goiás, Goiânia, Goiás, Brazil. The study was advertised through the use of electronic media (e-mails and student message groups) and through posters put up in the medical school during the month preceding the survey initiation. Data collection was based on a questionnaire created in Google Forms. The link to participate in the study was sent to all 656 students who were formally enrolled at that institute, with eligibility consisting of being ≥18 years of age. A total of 315 valid responses were received.

Data collected on participants' sociodemographic, behavioral, and clinical characteristics included age, sex, skin color, body mass index (BMI, kg/m^2^), living arrangements, marital status, employment status, means of enrolment in the university (open competition or through the quota system), current year of study (years 1-2 or 3-6), current smoker (yes/no), current alcohol or illicit drug consumption (yes/no), physical activity, chronic diseases, and history of physical or sexual abuse.

The way in which the student obtained entrance to the university was dichotomized into open competition or via the quota system in accordance with the model for entrance into public universities in Brazil. The course level was dichotomized into basic (years 1 or 2) or clinical (years 3-6) with respect to the 6-year duration of medical school in Brazil. The respondent was considered to be physically active if physical activity was performed for more than 150 minutes/week. A history of physical abuse was established from a positive answer to the question: “Have you ever suffered physical abuse?” (yes/no). A history of sexual abuse was determined from a positive response to the question: “Have you ever suffered sexual abuse?” (yes/no). A history of chronic disease was established from the question: “Do you have any chronic illnesses such as high blood pressure, asthma, hypothyroidism, or diabetes?” (yes/no).

### 2.2. Self-Report Questionnaires

#### 2.2.1. Anxiety

The Generalized Anxiety Disorder-7 (GAD-7) scale was used in its Brazilian Portuguese validated version [15]. This 7-item self-report questionnaire evaluates the presence of symptoms associated with anxiety over the preceding two weeks, including not being able to stop or control worrying. For each item, the presence of the symptom is rated from 0 to 3, with 0 meaning not at all, 1 on several days, 2 on more than half the days, and 3 nearly every day. The total score ranges from 0 to 21, with a score ≥ 10 being considered the cut-off for anxiety (http://www.phqscreeners.com/).

#### 2.2.2. Depression

The 9-item Patient Health Questionnaire (PHQ-9) was used. This self-report questionnaire is based on the diagnostic criteria for major depression listed in the Diagnostic and Statistical Manual of Mental Disorders, fourth edition (DSM-IV), and has been translated and validated for use in Brazilian Portuguese [16]. Questions deal with the presence of depressive symptoms over the preceding two weeks, including little interest or pleasure in doing things. Each item is scored from 0 to 3, with an overall severity score ranging from 0 to 27. A cut-off point of ≥10 was established for a diagnosis of depression (http://www.phqscreeners.com/).

#### 2.2.3. Suicidality

The Suicidal Behaviors Questionnaire-Revised (SBQ-R) was used to evaluate different domains of suicidality [17]. The SBQ-R has already been used in Brazilian Portuguese [18]. This 4-item questionnaire evaluates lifetime suicidal ideation and/or suicide attempts (1-4 points), the frequency of suicidal ideation over the preceding 12 months (1-5 points), the threat of a suicide attempt (1-3 points), and the self-reported likelihood of suicidal behavior in the future. An overall score ≥ 7 was considered the cut-off point for suicide risk in undergraduate students [17].

#### 2.2.4. Self-Efficacy

The general self-efficacy (GSE) scale has been validated for use in Brazil [19] and was used to evaluate self-efficacy beliefs. The GSE can be used in nonspecific contexts for the assessment of optimism and beliefs of personal competence to deal with a wide variety of stress-inducing situations. The scale consists of 10 questions, with answers based on a Likert-type scale (1 = not at all true, 2 = hardly true, 3 = moderately true, and 4 = exactly true). The mean score for the study population is used as the cut-off point for categorizing individuals as having greater or lower self-efficacy. In the present study, the mean score for the participants was 29. Therefore, a score ≤ 29 was considered indicative of low self-efficacy, while a score > 29 was considered to reflect high self-efficacy.

#### 2.2.5. Parental Bonding

The Parental Bonding Instrument (PBI) uses two scales termed care and overprotection or control to measure fundamental parental styles as perceived by the child regarding how they remember their parents during their first 16 years of life [20]. The PBI has been cross-culturally adapted for use in Brazilian Portuguese [21]. This 25-item questionnaire consists of 12 questions on care and 13 on overprotection. Care addresses statements such as “was affectionate to me” and “seemed emotionally cold to me.” Overprotection includes issues such as “let me decide things for myself” and “tried to make me dependent on him/her.” The measures are completed separately for both mothers and fathers. Specific scoring instructions were established for the PBI, with items being scored on a 4-point Likert-type scale (very like, moderately like, moderately unlike, and very unlike) ranging from 0 to 3; however, not all the items are scored in the same direction. Parents can be effectively assigned to one of four different parental bonding quadrants. Optimal parenting is characterized as high care and low overprotection; affectionate constraint as high care and high overprotection; neglectful parenting as low care and low protection; and affectionless control as low care and high overprotection [20]. The high and low categories are based on the following cut-off scores: a maternal/paternal care score of 27/24 and a maternal/paternal overprotection score of 14.5/12.5 [20].

### 2.3. Statistical Analysis

Results are presented as means and standard deviations (SD) or as absolute and relative frequencies according to the type of variable. Pearson's chi-square test was used to compare the prevalence of anxiety, depression, suicidal ideation, and low self-efficacy between the different parental bonding styles. Multiple logistic regression was used to determine the association between anxiety, depression, suicidal ideation, and low self-efficacy and parental bonding styles following adjustment for sociodemographic variables (model 1). An additional analysis was performed following adjustment for sociodemographic variables and depression to determine associations between anxiety, suicidal ideation, and low self-efficacy and parental bonding styles (model 2). In both models, odds ratios (OR) were calculated, together with their 95% confidence intervals (95% CI), considering optimal parenting as the reference category. Statistical significance was defined as *p* < 0.05. The internal consistency of the instruments used in the study was analyzed using Cronbach's alpha. The SPSS software package, version 21.0 (Armonk, NY: IBM Corp.), was used for the data analysis.

### 2.4. Ethics

The institution's internal review board approved the study protocol. All the students who participated in the study signed the electronic informed consent form included in the survey questionnaire.

## 3. Results

The study sample consisted of 315 medical students. Of these, 55.2% were male and 50.5% were White. The mean age was 23.35 ± 3.78 years (± SD), and the mean BMI was 23.73 ± 4.25 kg/m^2^. The majority were single (92.4%), lived with other people (77.1%), and did not work (83.2%). Overall, 58.7% of the students were in the clinical years (3-6) of medical school. Anxiety was found in 38.4% of the students, depression in 47.3%, and suicidal ideation in 36.2%. In addition, 44.1% of the students were found to have low levels of self-efficacy. Optimal parenting was the parenting style identified for 36.8% and 35.9% of participants in relation to their mothers and fathers, respectively (Table 1).

**Figure 1:**
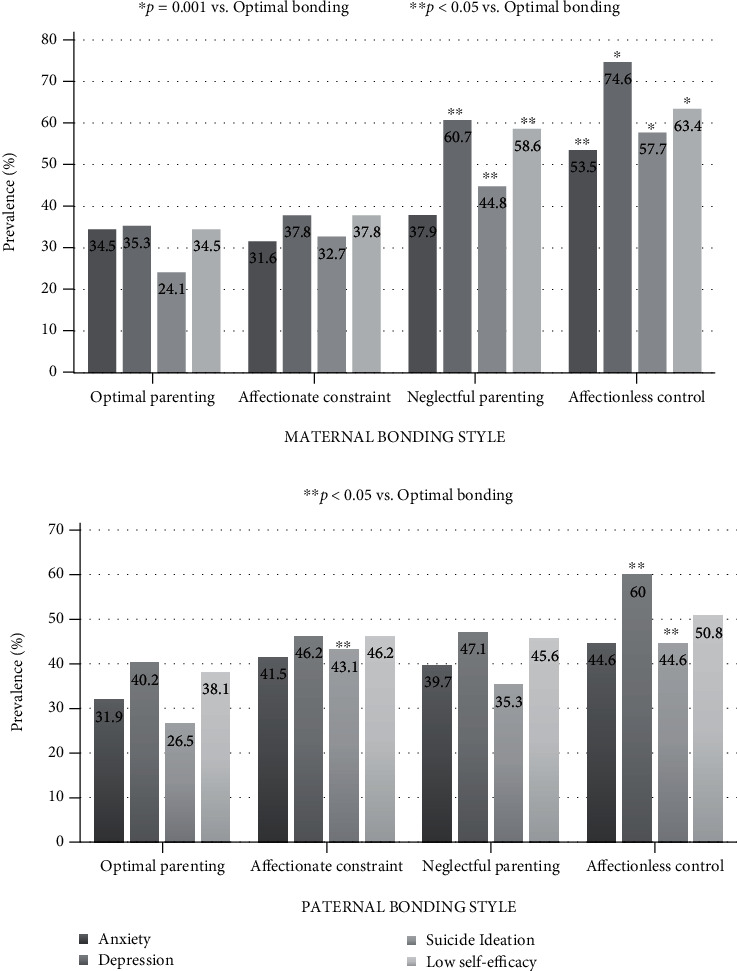
Anxiety, depression, suicidal ideation, and low self-efficacy according to the parental bonding quadrant.

In the group of students whose mothers were classified in the affectionless control and optimal parenting bonding quadrants, the prevalence rates were, respectively, 53.5% and 34.5% for anxiety (*p* < 0.05), 74.6% and 35.3% for depression (*p* = 0.001), 57.7% and 24.1% for suicidal ideation (*p* = 0.001), and 63.4% and 34.4% for low self-efficacy (*p* = 0.001). In relation to paternal bonding styles, the prevalence of depression for those in the quadrants affectionless control and optimal parenting was, respectively, 60.0% and 40.2% (*p* < 0.05). The prevalence of suicidal ideation for students whose paternal bonding styles were classified as affectionate constraint and optimal parenting was 43.1% and 26.5%, respectively (*p* < 0.05) (Figure 1).

Following adjustment for sociodemographic variables, the students in the maternal bonding quadrant affectionless control were significantly more likely than those in the optimal parenting quadrant to have anxiety (OR = 2.48; 95% CI: 1.15-5.33), depression (OR = 7.54; 95% CI: 3.2-17.18), suicidal ideation (OR = 3.62; 95% CI: 1.58-8.27), and low self-efficacy (OR = 3.81; 95% CI: 1.76-8.25). The students in the maternal bonding quadrant neglectful parenting were significantly more likely to have depression (OR = 3.24; 95% CI: 1.17-8.96). Following adjustment for sociodemographic variables, for the students in the parenting quadrant affectionate constraint in relation to fathers, the likelihood of suicidal ideation was significantly higher than that for the group in the optimal parenting quadrant (OR = 3.09; 95% CI: 1.36-3.02) (Table 2).

In the analysis adjusted for the sociodemographic variables and depression, however, no association was found between maternal or paternal bonding styles and anxiety (data not shown). Low self-efficacy remained positively associated with maternal affectionless control (OR = 2.61; 95% CI: 1.14 - 5.95) and suicidal ideation with paternal affectionate constraint (OR = 3.13; 95% CI: 1.35-7.27) (Table 3).

Cronbach's alpha was 0.889 for the GAD-7 scale, 0.888 for the PHQ-9, 0.872 for the SBQ-R, and 0.893 for the GSE scale. Internal consistency of the PBI was analyzed separately for the mother and the father according to its dimensions (care and overprotection). Cronbach's alpha for maternal care, maternal overprotection, paternal care, and paternal overprotection was 0.928, 0.863, 0.938, and 0.882, respectively.

## 4. Discussion

An association was found between dysfunctional parenting styles and anxiety, depression, suicidal ideation, and low self-efficacy in the group of medical students enrolled in the present study. This association differed as a function of the different maternal and paternal parenting styles. Following adjustment for a large number of potential confounders, some of the associations identified in the unadjusted analysis persisted. In particular, maternal, but not paternal, affectionless control remained strongly and persistently associated with depression and low self-efficacy beliefs. In addition, in the adjusted analysis, an association was found between paternal, but not maternal, affectionate constraint and suicidal ideation. To the best of our knowledge, this study is the first to demonstrate an association between parental bonding styles and general self-efficacy beliefs as well as an association between paternal affectionate constraint and suicidal ideation in medical students.

In the unadjusted analysis, the group of students whose mothers were classified in the affectionless control quadrant stood out from those whose mothers were in the other parenting style quadrants in whom the prevalence of anxiety, depression, suicidal ideation, and low self-efficacy was highest. With respect to paternal parenting styles, the frequency of depression and suicidal ideation was also high for those whose fathers were in the affectionless control quadrant. Coincidentally, earlier studies also highlight the interest of investigators from different countries in relation to the affectionless control parenting type [14, 22, 23]. This interest may be because affectionless control simultaneously encompasses the two criteria (low care and high overprotection) that are most harmful in the development of good social-emotional skills in children and adolescents [12]. Furthermore, the prevalence of depression, suicidal ideation, and low self-efficacy was also significantly higher among students whose mothers were classified in the neglectful parenting group, while the prevalence of suicidal ideation was significantly higher in those whose fathers were in the affectionate constraint quadrant. These findings are in agreement with those of other authors who have suggested the presence of at least one type of dysfunctional parental bonding style in association with mental disorders, suicidality, and models of the self and others [14, 22–24].

In the analysis adjusted for sociodemographic variables, the medical students whose mothers were in the affectionless control quadrant (low care and high overprotection) were strongly and significantly more likely to have depression compared to those in the maternal optimal parenting (high care and low overprotection) quadrant. In the maternal neglectful parenting group (low care and low protection), the risk of depression was greater compared to those whose mothers were in the optimal parenting group, but to a lesser degree. Nevertheless, no association was found between the parenting styles of the fathers and depression. These data are in agreement with the findings of other studies in which low maternal, but not paternal, care, with or without overprotection, was considered a predictor of depression [24–26]. Currently, the way children are raised has changed, with greater participation from fathers. However, the role of mothers as the caregiver remains predominant in various cultures and may perhaps partially explain this finding.

In relation to suicidal ideation following adjustment for sociodemographic variables, a significantly higher risk was found in the students in the maternal affectionless control group and in the paternal affectionate constraint group compared to the maternal and paternal optimal parenting groups, respectively. These findings are in agreement with the results of previous studies that highlighted the association between different types of dysfunctional parental bonding and suicidality [14, 27]. Previously, an association between dysfunctional bonding with the mother, but not with the father, and suicidal behavior has been reported [28]. Nevertheless, in the present study, the association between maternal bonding and suicidal ideation was no longer present following the inclusion of depression as a covariable. This finding suggests that depression affects the association between maternal affectionless control and suicidal ideation in medical students. In agreement with this finding, the role of depression in the association between dysfunctional bonding and suicidal behaviors has already been described [29]. Conversely, another study reported that a high level of maternal care was identified as a protective factor against suicidal ideation in medical students [30].

In the present study, the positive association between paternal affectionate constraint (high overprotection and high care) and suicidal ideation persisted even after adjustment for sociodemographic confounders and depression. This finding is compatible with results published by other investigators, who identified paternal attachment as the only significant predictive factor of suicidal behavior in a logistic regression model [31]. Nevertheless, there is a certain discrepancy between those results and the findings of other investigators, who reported an association between paternal high protection but low care and suicidality [32]. Paternal affectionate constraint has previously been associated with impaired formation of a positive internal working model of the self and others in male medical students and hospital staff [22]. The fact is that, despite particular and varying qualities, the importance of both maternal and paternal bonding for the biological and social development of children is undeniable. Whereas maternal bonding involves the biological conditions of pregnancy and breastfeeding, paternal bonding is shaped by social and cultural conditions. Even in current times, “in most societies, it is through paternal bonding that the child understands and takes her place within the family structure and also finds her place in the wider community” [33]. Cultural issues in Brazil may have played a role in the finding that having a father who, despite offering care, limits his child's autonomy with high overprotection resulted in a greater negative effect in the case of these medical students. Ambivalent behavior from the father, the figure of affection who would be expected to provide encouragement to face the challenges of the world, could have been responsible for the greater risk of suicidal ideation in the present study, and this is a subject that merits further attention in future studies.

Furthermore, the results of the present study show an independent and consistent association between maternal affectionless control and low self-efficacy beliefs in medical students. In a previous study conducted in five countries with 8,796 participants, general perceived self-efficacy appeared to be a universal construct which, at higher levels, was associated with different personality traits such as optimism, self-regulation, orientation towards the future, and self-esteem and at lower levels was associated with anxiety and depression [34]. A 10-year follow-up study of Norwegian physicians showed an association between lower self-esteem and adverse parental bonding [26]. In this respect, other authors have also shown an association between self-esteem and parental bonding in the general adult population [35]. In addition, maternal affectionless control has already been associated with a high degree of neuroticism and other personality vulnerabilities [23] in medical students and medical staff. Therefore, in agreement with the results of those earlier studies, low self-efficacy beliefs could perhaps be added to the list of susceptibility markers of personality traits associated with dysfunctional parental bonding, particularly maternal affectionless control.

Following adjustment for sociodemographic variables, only the students whose mothers were in the affectionless control quadrant were significantly more likely to have anxiety. Nevertheless, this association was no longer present following adjustment for depression. This finding could perhaps be explained by the existence of the comorbidity anxiety and depression (anxious depression), a relatively common condition in the literature [36]. Therefore, the current finding is to a certain extent in agreement with the earlier, nationally representative study conducted in the USA with 5,838 adolescents in which maternal low care and high protection were independently associated with anxiety and depression [24]. However, this finding is only partially in agreement with the results of other authors who reported significantly increased anxiety and depression in medical students in both parental bonding style groups, maternal and paternal affectionless control and optimal parenting [13]. This difference between the findings could possibly be explained by the different instruments used to investigate mental disorders and their respective cut-off points, as well as by the variables used in the multiple regression analysis and differences in the study population.

There is no doubt that the university environment, particularly that of medical school, is challenging, with numerous factors capable of triggering stress. Individuals who are better prepared psychologically should be emotionally protected when dealing with these challenges. A hostile family environment in childhood and adolescence, with adverse parental bonding styles, is relived in a segment of these students at medical school, an environment in which demands and expectations are high, with a clear and often emotionally cold hierarchy [37]. In the present study, following adjustment for sociodemographic data, variables related to student life, and events in the students' lives, an important association was found between distinct patterns of parental bonding styles and psychological issues in these medical students. Therefore, it is possible that preventive and therapeutic interventions related to the attachment theory, for example, attachment-based family therapy [38], family constellations [39], and therapy of bonding disorders and trauma [33], among others, could benefit medical students in their personal and academic career. These subject merits further investigation in future studies.

Certain limitations should be taken into consideration when analyzing the results of the present study. First, because of the cross-sectional design of the study, causality cannot be inferred. Secondly, the study involved self-reported measures, which could have generated a response bias. Nevertheless, the instruments applied in this study have been widely used and transculturally validated, including validation for use in Brazilian Portuguese. Furthermore, internal consistency was considered satisfactory for all the instruments used. Thirdly, the PBI is a tool for assessing an offspring's retrospective perception of his/her parents, which could to a certain extent compromise the answers obtained. However, the long-term stability and consistency of this instrument have already been validated [40]. Finally, the sample was restricted to medical students, thus limiting the generalization of these findings.

## 5. Conclusions

The results of the present study show independent and significant associations between maternal affectionless control and depression and low self-efficacy beliefs, between maternal neglectful parenting and depression, and between paternal affectionate constraint and suicidal ideation in medical students. These findings also suggest that depression affects the association between maternal affectionless control and anxiety and the association between maternal affectionless control and suicidal ideation. In light of these results, it would appear appropriate to take the attachment theory and its practical implications into consideration when treating medical students with mental disorders.

## Figures and Tables

**Table 1:** Characteristics of the undergraduate medical students (*n* = 315).

Variables	*N*	%
Sex		
Male	174	55.2
Female	141	44.8
Skin color		
White	159	50.5
Non-White	156	49.5
Living arrangement		
Lives with others	243	77.1
Lives alone	72	22.9
Marital status^∗^		
Without partner	291	92.4
With partner	22	7.0
Employment status		
Not working	262	83.2
Working	53	16.8
Admission to the medical course^∗^		
Open competition	174	55.2
Quota system	138	43.8
Undergraduate school year^∗^		
Basic (years 1-2)	129	41.0
Clinical (years 3-6)	185	58.7
Smoking		
Smoker	27	8.6
Nonsmoker	288	91.4
Alcohol consumption		
No	65	20.6
Yes	250	79.4
Illicit drug consumption		
No	230	73.0
Yes	85	27.0
Physical activity		
No	120	38.1
Yes	195	61.9
Chronic disease^∗^		
No	265	84.1
Yes	49	15.6
Physical assault		
No	282	89.5
Yes	33	10.5
Sexual abuse^∗^		
No	278	88.3
Yes	36	11.4
Anxiety		
No	194	61.6
Yes	121	38.4
Depression^∗^		
No	165	52.4
Yes	149	47.3
Suicide ideation		
<7 no	201	63.8
≥7 yes	114	36.2
Low self-efficacy		
Yes	139	44.1
No	176	55.9
Maternal bonding style^∗^		
Optimal parenting	116	36.8
Affectionate constraint	98	31.1
Neglectful parent	29	9.2
Affectionless control	71	22.5
Paternal bonding style^∗^		
Optimal parenting	113	35.9
Affectionate constraint	65	20.6
Neglectful parent	68	21.6
Affectionless control	65	20.6
Quantitative variables		Mean (SD)
Age (years)	18.0–41.0	23.35 ± 3.78
Body mass index (kg/m^2^)	16.3–57.0	23.73 ± 4.25

^∗^The frequency of the students in the different categories differs due to missing values.

**(a):** 

Parental bonding style	*N*	Anxiety					Depression				
		*n*	Crude OR (95% CI)	*p* value	Adjusted OR (95% CI)	*p* value	*n*	Crude OR (95% CI)	*p* value	Adjusted OR (95% CI)	*p* value
Mother											
Optimal parenting	116	40	1.00 (ref)	—	1.00 (ref)	—	41	1.00 (ref)	—	1.00 (ref)	—
Affectionate constraint	98	31	0.88 (0.50-1.56)	0.659	0.89 (0.46-1.74)	0.746	37	1.11 (0.63-1.94)	0.715	1.13 (0.59-2.17)	0.704
Neglectful parenting	29	11	1.16 (0.50-2.70)	0.728	0.72 (0.23-2.22)	0.568	17	2.83 (1.21-6.61)	0.016^∗^	3.24 (1.17-8.96)	0.024^∗^
Affectionless control	71	38	2.19 (1.20-4.00)	0.011^∗^	2.48 (1.15-5.33)	0.020^∗^	53	5.39 (2.79-10.38)	0.001^∗^	7.54 (3.20-17.78)	0.001^∗^
Father											
Optimal parenting	113	36	1.00 (ref)	—	1.00 (ref)	—	45	1.00 (ref)	—	1.00 (ref)	—
Affectionate constraint	65	27	1.52 (0.81-2.86)	0.195	1.78 (0.87-3.69)	0.117	30	1.28 (0.69-2.37)	0.438	1.56 (0.77-3.14)	0.215
Neglectful parenting	68	27	1.41 (0.75-2.64)	0.284	1.89 (0.89-4.00)	0.094	32	1.32 (0.72-2.43)	0.366	1.75 (0.87-3.53)	0.117
Affectionless control	65	29	1.72 (0.92-3.23)	0.090	1.64 (0.73-3.67)	0.229	39	2.23 (1.20-4.17)	0.012^∗^	2.06 (0.93-4.60)	0.076

**(b):** 

Parental bonding style	*N*	Suicide ideation					Low self-efficacy				
		*n*	Crude OR (95% CI)	*p* value	Adjusted OR (95% CI)	*p* value	*n*	Crude OR (95% CI)	*p* value	Adjusted OR (95% CI)	*p* value
Mother											
Optimal parenting	116	28	1.00 (ref)	—	1.00 (ref)	—	76	1.00 (ref)	—	1.00 (ref)	—
Affectionate constraint	98	32	1.52 (0.84-2.77)	0.168	1.37 (0.68-2.80)	0.381	61	1.15 (0.66-2.02)	0.619	1.18 (0.62-2.21)	0.615
Neglectful parenting	29	13	2.55 (1.09-5.95)	0.030^∗^	1.95 (0.65-5.80)	0.230	12	2.69 (1.17-6.19)	0.020^∗^	2.65 (0.97-7.19)	0.057
Affectionless control	71	41	4.29 (2.28-8.10)	0.001^∗^	3.62 (1.58-8.27)	0.002^∗^	26	3.29 (1.78-6.09)	0.001^∗^	3.81 (1.76-8.25)	0.001^∗^
Father											
Optimal parenting	113	30	1.00 (ref)	—	1.00 (ref)	—	70	1.00 (ref)	—	1.00 (ref)	—
Affectionate constraint	65	28	2.09 (1.09-3.98)	0.025^∗^	3.09 (1.36-7.02)	0.007^∗^	35	1.40 (0.75-2.59)	0.291	1.38 (0.67-2.84)	0.383
Neglectful parenting	68	24	1.51 (0.79-2.89)	0.214	1.72 (0.80-3.71)	0.168	37	1.36 (0.74-2.51)	0.319	1.51 (0.75-3.01)	0.244
Affectionless control	65	29	2.23 (1.17-4.24)	0.015^∗^	1.37 (0.60-3.16)	0.464	32	1.68 (0.91-3.11)	0.100	1.28 (0.61-2.70)	0.513

OR: odds ratio; CI: confident interval. Multiple logistic regression adjusted for age, sex, body mass index, skin color, marital status, living arrangement, employment status, process for admission to medical school, undergraduate school year, smoking, alcohol consumption, illicit drug consumption, physical activity, chronic disease, physical abuse, and sexual abuse.

**Table 3:** Association between anxiety, suicidal ideation, and low self-efficacy according to maternal and paternal bonding styles following adjustment for sociodemographic variables and depression (model 2).

Parental bonding style	*N*	Suicide ideation					Low self-efficacy				
		*n*	Crude OR (95% CI)	*p* value	Adjusted OR (95% CI)	*p* value	*n*	Crude OR (95% CI)	*p* value	Adjusted OR (95% CI)	*p* value
Mother											
Optimal parenting	116	28	1.00 (ref)	—	1.00 (ref)	—	76	1.00 (ref)	—	1.00 (ref)	—
Affectionate constraint	98	32	1.52 (0.84-2.77)	0.168	1.36 (0.65-2.87)	0.420	61	1.15 (0.66-2.02)	0.619	1.15 (0.61-2.19)	0.670
Neglectful parenting	29	13	2.55 (1.09-5.95)	0.030^∗^	1.41 (0.45-4.34)	0.548	12	2.69 (1.17-6.19)	0.020^∗^	1.95 (0.67-2.85)	0.221
Affectionless control	71	41	4.29 (2.28-8.10)	0.001^∗^	1.96 (0.80-4.83)	0.142	26	3.29 (1.78-6.09)	0.001^∗^	2.61 (1.14-5.95)	0.023^∗^
Father											
Optimal parenting	113	30	1.00 (ref)	—	1.00 (ref)	—	70	1.00 (ref)	—	1.00 (ref)	—
Affectionate constraint	65	28	2.09 (1.09-3.98)	0.025^∗^	3.13 (1.35-7.27)	0.008^∗^	35	1.40 (0.75-2.59)	0.291	1.24 (0.67-2.64)	0.571
Neglectful parenting	68	24	1.51 (0.79-2.89)	0.214	1.60 (0.72-3.54)	0.246	37	1.36 (0.74-2.51)	0.319	1.37 (0.65-2.85)	0.406
Affectionless control	65	29	2.23 (1.17-4.24)	0.015^∗^	1.18 (0.49-2.86)	0.713	32	1.68 (0.91-3.11)	0.100	1.18 (0.53-2.61)	0.687

OR: odds ratio; CI: confident interval. Multiple logistic regression adjusted for age, sex, body mass index, skin color, marital status, living arrangement, employment status, process for admission to medical school, undergraduate school year, smoking, alcohol consumption, illicit drug consumption, physical activity, chronic disease, physical abuse, sexual abuse, and depression.

## Data Availability

The data used to support the findings of this study are included within the article.
